# The Association Between Refractive Errors and Breastfeeding in Pakistani Children: A Case-Control Study

**DOI:** 10.7759/cureus.28311

**Published:** 2022-08-23

**Authors:** Malab S Balouch, Muhammad Shahbaz, Mohammad M Balouch, Mechale S Balouch, Muneeb U Abbasi

**Affiliations:** 1 Neurorehabilitation Unit, Charing Cross Hospital London, Imperial College Healthcare Trusts, London, GBR; 2 Department of Optometry, Basheeran Umar Eye Hospital, Islamabad, PAK; 3 Ayub Medical College, Ayub Teaching Hospital, Abbottabad, PAK; 4 Department of Radiology, Islamabad Diagnostics Center, Islamabad, PAK; 5 Department of Ophthalmology, Combined Military Hospital, Rawalakot, PAK

**Keywords:** pakistan, vision, childhood nutrition, myopia, errors of refraction, refractive errors, breastfeeding

## Abstract

Objective

The aim of this study was to determine the association between breastfeeding and the occurrence of refractive errors (REs) among children.

Methodology

This was a (retrospective) case-control study carried out between December 1, 2021, and March 30, 2022, at the Basheeran Umar Eye Hospital in Islamabad, Pakistan, and Sheikh Khalifa bin Zayed Al Nayhan Combined Military Hospital in Rawalakot in Kashmir, Pakistan. A total of 200 participants between the ages of five and 16 years (106 cases with REs and 94 controls without REs) were included in this study. After obtaining informed consent from the parent accompanying the participant, the parent was further interviewed to extract information regarding biodata, breastfeeding, and other parameters including parental myopia, the number of hours spent on outdoor activities, on gadgets, and doing near work; this data was entered into a questionnaire. The participant’s visual acuity was then checked using the Snellen chart. Data were analyzed using SPSS Statistics version 20.0 (IBM Corp., Armonk, NY) and statistical tests such as multivariate regression analysis and chi-square were carried out and odds ratios (OR) were calculated.

Results

There were 74 (37%) females and 126 (63%) males, with 67.5% residing in urban localities and only 32.5% hailing from rural areas. Testing revealed no significant association between REs and area of residence (p=0.97) or the gender of the participant (p=0.74). Hypermetropia was the most common RE among our participants (59.4%).

Breastfeeding was significantly associated with errors of refraction (OR: 27.852, 95% CI: 3.686-210.45, χ^2^=21.680, p<0.001, degrees of freedom: 1), and we observed a lower risk of REs in children who had been breastfed. There was no association between REs and the number of hours spent outdoors per week or the number of hours spent using gadgets per week. However, there was a significant association between the development of REs and the duration of breastfeeding and the number of hours spent on near work.

Conclusion

This study demonstrates that breastfeeding plays a protective role in the prevention of REs. The type of breastfeeding also had a significant effect on the development of REs, i.e., children exclusively breastfed (regardless of the duration of breastfeeding) were less likely to develop errors of refraction in the future.

## Introduction

According to the World Health Organization (WHO), refractive errors (REs) refer to the inability of one's eye to focus images on the retina, leading to the blurring of vision and, in severe cases, functional blindness [1]. Approximately 2.8 billion people suffer from REs worldwide. While 80% of visual disability is avoidable [2], only 1.8 million are fortunate enough to have access to eye examinations and correction materials; the remaining 500 million, mostly residing in developing countries, are forced to live with uncorrected errors [3].

Researchers have long been trying to find associations between REs and various factors; these include a family history of REs [4,5], prolonged near-work activities like studying, watching television, or the use of computers [6-8], as well as systemic ophthalmic diseases [9]. Some studies even suggest a relationship between the development of errors of refraction and low birth weight and the race of the child [9-11]. Parental myopia is perhaps the most research-backed and clinically recognized risk factor for a child developing myopia, followed by outdoor activity as a protective element in environmental factors [4,5,11,12,13]. Studies have also been carried out to investigate the effects of breastfeeding on visual development in the early years of life; these have revealed that breast milk contains omega-3 and essential fatty acids that play a vital role in cognitive and visual development [14], reducing various ocular morbidities, and preventing retinopathy of prematurity [15]. Bozkurt et al. have gone as far as calling it the "miracle food" in reducing ocular diseases, and it plays a vital role in visual development, particularly stereopsis [16].

Chong et al. conducted a study in Singapore that demonstrated significantly lower myopia in breastfed children [17]. While some researchers claim that breastfeeding is related to hypermetropia only, others such as Rudnicka et al. and Shirzadeh et al. have established no relationship between breastfeeding and REs [15,18,19].

Poor vision affects a child’s life experiences and motor development, besides restricting academic progress due to the delay in absorbing new information; all of these truly affect the ability to perform at school or any other social situations [3,20]. Visual problems not only have adverse effects on the physical, educational, and psychological well-being of the child but also negatively affects social development, as the child is unable to pick up on non-verbal communication by peers and mentors at a very young age [3,21]. By rectifying uncorrected REs, we can radically improve the quality of life and education for many people [3,22].

The aim of our research is to determine the association between breastfeeding and the occurrence of REs. Uncorrected REs are a major cause of functional blindness in Pakistan and other developing countries. It can even eventually lead to blindness in some cases, which is a heavy and avoidable cost to pay since visual acuity tests can be performed very easily and quickly.

## Materials and methods

This was a case-control (retrospective) study carried out between December 1, 2021, and March 30, 2022, at the Basheeran Umar Eye Hospital in Islamabad and Sheikh Khalifa bin Zayed Al Nayhan Combined Military Hospital in Rawalakot in Kashmir, Pakistan. A total of 200 participants between the ages of five and 16 years were included in the study, out of which 106 were cases and the remaining 94 controls. Due to a narrow time frame, convenience non-probability sampling was used.

Cases were defined as individuals with REs (visual acuity of 6/9 or worse in at least one eye), and controls were participants without REs. The inclusion criteria were as follows: children between five and 16 years of age, with parental consent for inclusion in the study; the exclusion criteria were as follows: children with acute or chronic eye diseases that may affect visual acuity and those whose parents refused to consent.

Approval was obtained from the hospital’s ethics committee (the Research Ethics Committee of Sheikh Khalifa Bin Zayed Al Nayhan Combined Military Hospital Rawalakot; approval number: RTMC# MED-2020-296-16265). Additionally, the subjects' parents were verbally informed about the aim of our questionnaires and research and then informed verbal consent was taken prior to proceeding with the interview and visual acuity check.

The data were collected using questionnaires as a data collection tool and the parents of the subjects were interviewed to extract information regarding biodata, breastfeeding (duration and type of breastfeeding, i.e. exclusively, mostly, partly, or never breastfed), and other parameters including parental myopia, the number of hours spent on outdoor activities, using gadgets, and on near-work activities such as reading or arts and crafts. In participants who were not exclusively breastfed, the use of alternative sources, if any, was noted (formula/cow milk/other). The subjects were taken into the side room to measure their visual acuity using a Snellen eye chart, and their refraction test was done by the optometrist (except when a medical student was collecting data). As data was being collected from two different institutions, it was done via Google Docs. We used the Snellen chart due to its easier availability in the ophthalmology outpatient department as well as wards. However, for the ease of data entry and analysis in our statistical software, the visual acuity was converted from Snellen to LogMar to accommodate for decimals.

Data were analyzed using SPSS Statistics version 20.0 (IBM Corp., Armonk, NY) and statistical tests such as multivariate regression analysis and chi-square were carried out and odds ratios (OR) were calculated. The Results and Discussion sections have been prepared in accordance with this analysis.

## Results

There was a total of 200 participants, with 106 cases (53%) and 94 controls (47%); 63% (126) of our participants were male and only 37% (74) were females, with 67.5% residing in urban localities and only 32.5% in rural. Testing revealed no significant association between REs and area of residence (p=0.97) or the gender of the participant (p=0.74). Out of the 106 participants with REs, hypermetropia had a higher prevalence than myopia, with 59.4% (63) and 40.6% (43) of our participants having hypermetropia and myopia respectively. The average age at the diagnosis of RE was 6.7 years (SD: 2.21).

Our study demonstrated that breastfeeding was indeed significantly associated with REs (OR: 27.85, 95% CI: 3.69-210.45, χ2=21.680, p<0.001, degrees of freedom: 1). Breastfeeding during the first few years of life had a protective effect on vision, and it decreased the likelihood of developing REs. Further details are presented in Table 1.

**Table 1:** The relationship between breastfeeding and visual impairment Odds ratio: 27.852; 95% CI: 3.686-210.45

		Vision		Total
		Normal	Impaired	
Breastfeeding of the child	Yes	94	81	175
		53.70%	46.20%	100.00%
	No	1	24	25
		4.00%	96.00%	100.00%
Total		95	105	200
		47.50%	52.50%	100.00%

Surprisingly, nearly 87% of the participants were breastfed, with the average duration of breastfeeding being 16.2 months (SD: 9.92); 55.5% of children were exclusively breastfed, 13.5% mostly, and 18.5% were at least partly breastfed (Table 2). There was a significant relationship between the type of breastfeeding (exclusively/mostly/partly breastfed) and the protective effect it would have on vision (χ2=56.79, DF: 3, p<0.001), with children who were exclusively breastfed having the best visual outcomes. In children who were not breastfed or were partly breastfed, the most popular milk substitute was formula milk (29%), and there appeared to be a significant association between the type of milk given and the chances of developing RE too (χ2=18.94, DF: 3, p<0.001).

**Table 2:** Frequencies of various types of breastfeeding

Type of breastfeeding	Frequency	Percentage
Never breastfed	25	12.5
Exclusively breastfed	111	55.5
Mostly breastfed	27	13.5
Partly breastfed	37	18.5
Total	200	100.0

Children were spending an average of 23.9 hours per week doing near-work activities such as reading, homework, and arts and crafts; an average of 28.8 hours per week were spent on outdoor activities and 8.6 hours on using gadgets such as mobile phones, computers, and television (Table 3). Multivariable regression analysis (Wald test) was used to determine the statistical significance for each of the independent variables (duration of breastfeeding, number of hours spent outdoors per week, hours spent doing near work per week, and the number of hours spent using gadgets per week) on the dependent variable (RE in participant). From the results, we can see that the duration of near work (p=0.024) and the duration of breastfeeding (p=0.044) had a significant effect on the development of REs, but outdoor activity (p=0.997) and gadget use (p=0.099) did not add significantly to the development of REs. As the coefficients of the near-work duration and duration of breastfeeding were negative, it means that they had a negative association with REs, thereby playing a protective role toward the vision. However, on performing regression analysis, it appeared that the duration of breastfeeding had no significant effect on the severity of errors of refraction. The strongest determinant in the development of REs (especially myopia) was parental myopia (χ2=88.17, p<0.001).

**Table 3:** Frequencies of various activities

	Minimum	Maximum	Mean	Std. deviation
Age of participant (years)	4	16	8.85	2.291
Visual acuity of the worse eye (LogMar)	0.00	1.30	0.3528	0.40270
Age at which refractive error was diagnosed (years)	0	15	3.93	3.687
Duration of breastfeeding in months	0.00	30.00	16.2460	9.92634
The number of hours spent outdoors per week	4	60	28.79	14.248
The number of hours spent doing near-work activity per week	0	60	23.93	14.191
The number of hours spent using mobile phone/computer/television per week	2	30	8.60	4.446

A vast majority of the participant's mothers were graduates (Table 4); however, maternal education did not have an effect on the duration of breastfeeding of the child. Levene test revealed that the education subgroups were not homogeneous (p=0.002), and hence we performed the Kruskal-Wallis test, which demonstrated no significant difference in the duration of breastfeeding based on the level of maternal education (p=0.774).

**Table 4:** Maternal education levels

Level of education	Frequency	Percentage
Primary	15	7.5
Middle	15	7.5
Matric	36	18.0
Intermediate	57	28.5
Graduate	77	38.5
Total	200	100.0

## Discussion

REs appear in childhood and continue through adult life; unfortunately, our society does not pay much attention to them and due to the lack of visual examination at preschool levels for children, these errors often remain undiagnosed for decades [4]. Statistical data regarding the prevalence of REs in children globally as well as in Pakistan is relatively scarce; nonetheless, various researchers have attempted to estimate it in their regions. The most recent survey was carried out in 2017, at CMH Mangla, where 9.4% of the participants had REs, with more females (53.6%) affected as compared to males (46.6%) [5]. Similarly, a study at Lakki Marwat in Khyber Pakhtunkhwa in 2013 demonstrated that 5.4% of the children suffered from REs, 88.6% of which were uncorrected, and females were more affected than males at 6.8% and 5.0% respectively [23]. While Ali et al. reported figures of 19.8% in 2007 at the Punjab Institute of Preventative ophthalmology in Lahore (43% myopic and 21.5% hyperopic), Alam et al. documented a figure of 10.9% in Karachi [4,6]. Unlike the above-mentioned studies, REs were more common in our male participants (61.3%) than females (55.4%), and a higher proportion of participants had hypermetropia (59.4%) than myopia (40.6%). This clearly indicates the variation in collected data among different parts of Pakistan.

The main aim of our research was to investigate the relationship between breastfeeding and REs, and our study demonstrated that breastfeeding did in fact have a protective effect on vision (χ2=21.680, p<0.001), although duration and type of breastfeeding did not significantly affect the visual outcome of the participants. Previous research on the biochemistry of breast milk suggested that its constituents, such as polyunsaturated fatty acids and anti-oxidants have an effect on early neural development, thereby influencing the development of the retina and growth of the eye [24]. This could potentially explain the protective effect of breastfeeding on vision. Researchers have claimed that the retina (and crystalline lens) is protected from oxidative damage by lutein and zeaxanthin, which are present more densely in the macula. They cannot be synthesized by the infant and must be attained via their diet, and it was observed that serum lutein levels are much higher in breastfed children than in formula-fed ones [17].

Although limited research has been done on the protective effects of breastfeeding on vision, all studies have yielded varying results. Sham et al. (2009) [25] concluded that the breastfeeding status and duration of breastfeeding were independently associated with REs, and children with a history of breastfeeding were found to be more hyperopic (having a more positive spherical equivalent) than those who were not breastfed. On the other hand, studies by Rudnicka et al. (2008) [18], Shirzadeh et al. (2016) [19], and Owen et al. (2018) [26] revealed no relationship between infant breastfeeding and future visual development; instead, it was implied that it was more influenced by other environmental factors such as birth weight, age, parental education, and maternal age. It is essential to highlight that our study found no association between gender, area of residence, or maternal education on the development of REs. We also investigated other factors like the time the child was spending on outdoor activities and the duration of gadget use and carrying out near-work tasks such as reading and writing. Testing revealed that while the former two did not have an effect on vision, surprisingly, near-work was protective of vision. Similar to our results, a very extensive study by Chong et al. [17] revealed a reduced risk of myopia development in breastfed children (43% lower than non-breastfed). Liu et al. [24] had similar findings and demonstrated that children breastfed for a duration of more than six months had a 49.8% lower chance of developing myopia and that parental myopia was strongly associated with the child developing myopia, which was also highlighted in our results [12,13,24,25].

Of note, 87% of the children participating in our study were breastfed, which is a little higher compared to other studies, possibly due to breastfeeding being highly promoted in our culture. The average duration of breastfeeding was 16.2 months, and 55.5% of the participants were exclusively breastfed, unlike in Sham et al.’s [25] study where only 14% were breastfed. This can again be explained by cultural differences. Unlike our study, they confirmed that the duration of breastfeeding did have an effect on the development of REs while the type of breastfeeding did not. Nonetheless, exclusively breastfed children had higher mean refraction (although not significant). However, our findings revealed the opposite as our data showed that the type of breastfeeding (exclusive/mostly/partly/never breastfed) did have a significant effect on vision, with exclusively breastfed children being least likely to develop REs; however, no significant association was seen between the duration of breastfeeding and vision/REs. Maternal education was not associated with the duration of breastfeeding either.

The relationship between near-work activity and the development of REs is variable in the previous studies. Our data revealed that near work was protective of vision; on the other hand, Mutti et al. [8] and Jones et al. [11] noted no such association in their work. O’Donoghue et al. [12] noted an increased risk of myopia with increased near-work activity but their findings were not significant. It is likely that this large variation in the association between near work and vision could be due to the rather crude methods of establishing time spent on various near-work activities, and questionnaires are unlikely to provide accurate data on it due to recall bias. The same applies to the association between time spent outdoors and its effects on vision. Although most studies show a positive relationship between outdoor time and vision, our data revealed no such association [9,12,13].

Although our study revealed a statistically significant relationship between REs and breastfeeding, it has its limitations, and more in-depth research needs to be carried out to identify various aspects related to breastfeeding including maternal nutrition and smoking. Other factors such as the participant’s birth weight and general nutritional status during childhood must also be incorporated into the research. Further investigation into the biochemistry of human milk is required as most of the studies were unable to identify the exact constituent to be credited for the beneficial effects of breast milk. Similarly, for mothers who are unable to breastfeed, an additional exploration into the constituents of the alternative forms of milk is essential.

A more precise and accurate method of measuring the time spent on various activities such as studying, outdoor activities, and gadget use needs to be devised. As most parents in our study were oblivious to the amount of time their children spent on these activities, our data suffered due to recall bias. This could be avoided in better-funded setups where more advanced technology such as smart watches/gadgets could be used to measure these parameters in prospective studies, ideally with larger sample sizes.

## Conclusions

Based on our findings, breastfeeding plays a protective role in the prevention of REs. The type of breastfeeding also had a significant effect on the development of REs, i.e., children who were exclusively breastfed were less likely to develop REs in the future; however, the duration of breastfeeding did not have an effect on the vision. The amount of time spent outdoors and on the use of gadgets did not significantly affect the visual outcome of a child in terms of errors of refraction; however, near-work activities such as reading appeared to have a protective effect on vision. REs were much more common in male participants than females, and in individuals residing in urban than those in rural settings.
